# Corneal Reinnervation and Sensitivity Recovery after Pterygium Excision

**DOI:** 10.1155/2020/1349072

**Published:** 2020-02-22

**Authors:** ZhanLin Zhao, JiaYing Zhang, Hong Liang, SiYi Zhang, ChunYi Shao, XianQun Fan, Yao Fu

**Affiliations:** ^1^Department of Ophthalmology, Ninth People's Hospital, Shanghai JiaoTong University School of Medicine, Shanghai, China; ^2^Shanghai Key Laboratory of Orbital Diseases and Ocular Oncology, Shanghai, China; ^3^Department of Ophthalmology III, Quinze-Vingts Hospital, DHU Sight Restore, INSERM-DHOS CIC, and Vision Institute, INSERM, U968, Sorbonne Universités, Paris, France

## Abstract

**Purpose:**

To evaluate changes in corneal sensitivity and subbasal nerve density after pterygium excision.

**Methods:**

This prospective trial included 22 eyes with nasal primary pterygium and 18 controls. Corneal sensitivity was evaluated using a Cochet–Bonnet esthesiometer in the nasal, superior, temporal, inferior, and center quadrants of the cornea before surgery and 10 days, 1 month, and 3months after surgery. The central cornea was analyzed using *in vivo* confocal microscopy (IVCM) before surgery and 1 and 3 months after surgery. Subbasal nerve density and other nerve parameters were analyzed using NeuronJ. Nerve tortuosity was evaluated and graded in individual IVCM scans. The tear film break-up time (TBUT) test and Schirmer's test were performed before surgery, as well as 1 and 3 months after surgery. All the same tests were performed in the controls.

**Results:**

All affected eyes showed a significant increase in corneal sensitivity in the nasal corneal quadrant after surgery when compared with preoperative data (*F* = 37.3; *P* < 0.01). Compared with controls, pterygium patients demonstrated decreased corneal subbasal nerve density (*P* < 0.01). Compared with controls, pterygium patients demonstrated decreased corneal subbasal nerve density (*P* < 0.01). Compared with controls, pterygium patients demonstrated decreased corneal subbasal nerve density (*P* < 0.01). Compared with controls, pterygium patients demonstrated decreased corneal subbasal nerve density (*F* = 37.3; *P* < 0.01). Compared with controls, pterygium patients demonstrated decreased corneal subbasal nerve density (*P* < 0.01). Compared with controls, pterygium patients demonstrated decreased corneal subbasal nerve density (*F* = 37.3; *P* < 0.01). Compared with controls, pterygium patients demonstrated decreased corneal subbasal nerve density (

**Conclusion:**

Pterygium patients demonstrated deteriorated corneal subbasal nerve fibers when compared with healthy controls in terms of nerve length, nerve trunks, and nerve branches. Therefore, pterygium excision improves corneal sensitivity and increases corneal subbasal nerve density.

## 1. Introduction

Pterygium is a wing-shaped fibrovascular growth from the bulbar conjunctiva extending toward the cornea. It is a common chronic inflammatory disease of the ocular surface, with a prevalence of 9.84% in China [1], and it involves symptoms of ocular irritation and visual disturbance. Chronic exposure to ultraviolet light plays a key role in the pathogenesis of pterygium by damaging both limbal stem cells and the corneal nerve plexus [2–4], leading to the release of sensory neuropeptides [5–7] that induce the characteristic centripetal proliferation and migration of corneal epithelial cells and fibroblasts, as well as angiogenesis in the conjunctiva [8–11].

Pterygium can impact the ocular surface in a variety of ways. In one study, patients with pterygium had lower Schirmer's test results and tear film stability [12], and tear film break-up times (TBUTs) have been found to increase significantly after primary pterygium excision [13, 14]. Furthermore, hypoesthesia has been reported in the nasal corneas of patients with pterygium [15, 16]. In this regard, corneal sensitivity is a measure of corneal nerve function and indicates the integrity of the protective mechanisms of the ocular surface. Partial or complete corneal anesthesia may indicate neural damage before any lesions can be detected [17], and it has been associated with ensuing epithelial breakdown and neurotrophic keratitis [18].

The cornea is a highly innervated structure, deriving its sensory nerve supply from the trigeminal nerve and the less numerous sympathetic and parasympathetic nerve fibers, which play an important role in sensitivity, protection, and nutrition [19]. Although the morphologic appearance of the corneal nerves in ocular surface diseases has been widely studied using *in vivo* confocal microscopy (IVCM) [20], results differ as to the relationship between the corneal nerve structures and corneal nerve function [17, 21, 22]. Two studies showed that both epithelial membrane and Bowman's membrane are altered in the affected corneas in pterygium patients whose nerve plexus could not be detected using *in vivo* laser scanning confocal microscopy [23, 24].

Currently, the clinical criteria for evaluating recovery after pterygium excision mainly aim to distinguish between regrowth and healing of the lesion. Clinically, complete recovery is usually achieved by 1 month after pterygium excision [25]. Even though sensitivity is decreased in pterygium-affected corneas [15, 16], no previous investigations have studied how corneal sensitivity is related to lesions in the corneal nerve fibers in patients with pterygium. Moreover, several studies have reported that corneal sensitivity recovers before reinnervation after corneal surgery [26, 27]. Therefore, in the present study, we investigated both the corneal subbasal nerve plexus and corneal sensitivity to determine whether functional changes are related to any anatomical modification.

## 2. Materials and Methods

This prospective, nonrandomized, self-controlled trial included 17 patients (22 eyes) with primary nasal pterygium and 18 healthy controls (18 eyes). We excluded subjects with a history of ocular surgery, ocular trauma, infectious keratitis, severe dry eye symptoms (tear film break-up time < 5 s and Schirmer's test < 10 mm), use of contact lenses, use of topical treatments with known corneal toxicity, including antiglaucoma drugs or NSAIDs, presence of Sjögren's syndrome or chronic neuropathy, facial paralysis, or diabetes mellitus, and other systemic diseases that can affect the ocular anterior segment.

Pterygium was graded according to the system described by Benitez-del-Castillo et al. [28], which is as follows: grade T1, episcleral vessels unobscured by pterygium body; grade T2, partially obscured episcleral vessels; and grade T3, episcleral vessels obscured by the pterygium body. All patients enrolled in the present study had grade T3 pterygium.

This study was approved by the Investigational Review Board of Shanghai Ninth People's Hospital, Shanghai Jiaotong University School of Medicine, Shanghai, China (approval number: 2018-21-T21). The research followed the tenets of the Declaration of Helsinki. All subjects were informed about the aim of the study, and informed consent was obtained from each patient.

### 2.1. Surgical Procedures

All surgeries were performed by the same team using the same technique [29]: excision of the pterygium followed by a free limbal-conjunctival autograft taken from a superior position. After surgery, all patients received an identical regimen of topical levofloxacin eye drops (Santen Pharmaceutical, Japan), 0.1% fluorometholone eye drops (Santen Pharmaceutical, Japan), and 0.5% sodium hyaluronate eye drops (Santen Pharmaceutical, Japan), which were tapered off over 1 month. The nylon sutures were removed between day 7 and day 10. Clinical measurements were performed as described in the following.

### 2.2. Slit-Lamp Biomicroscopy

Color images of each eye with pterygium were acquired using a slit-lamp-mounted digital camera system (Topcon SL-D digital slit-lamp; Topcon, Tokyo, Japan) before surgery (preop) and 10 days (10 d postop), 1 month (1 m postop), and 3 months (3 m postop) after surgery. A 16X magnified image was taken using a 45-degree angled beam of white light projected through a diffusion filter. The region of interest was identified on the photograph. The area of pterygium extension onto the cornea from the limbus as well as the area of the cornea itself were quantified using the polygon selection tool and the analyze/measure command of ImageJ analysis software (W Rasband, National Institutes of Health, Bethesda, MD; http://rsb.info.nih.gov/ij/). The pterygium area was later calculated as a percentage of the corneal area.

### 2.3. Tear Film Break-Up Time Test

The TBUT was measured using sodium fluorescein strips (Jing Ming, Tianjin, China); the average of two consecutive break-up times was calculated in healthy controls and in patients before surgery as well as 1 and 3 months after surgery.

### 2.4. Schirmer's Test

As the final step in the examination, Schirmer's test was performed without topical anesthesia by placing a standard paper strip in the midlateral portion of the lower fornix. The amount of wetting was recorded after 5 minutes. Patients were asked to blink normally during the test. This test was performed in healthy controls and in patients before surgery as well as 1 and 3 months after surgery.

### 2.5. Corneal Sensitivity Measurement

**Figure 1:**
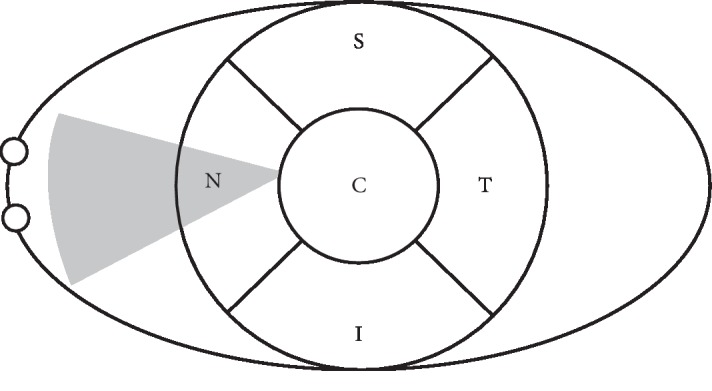
Eye diagram depicting different locations tested using the Cochet–Bonnet esthesiometer. The gray triangle represents the pterygium before surgery.

Corneal sensitivity was measured using the Cochet–Bonnet esthesiometer (Luneau Ophthalmlogie, Chartres, France) before surgery as well as 10 days, 1 month, and 3 months after surgery in both patients and healthy controls. The Cochet–Bonnet esthesiometer activates mechanical and polymodal nociceptors, which represent about 90% of all corneal nociceptors [30]. The superior, inferior, temporal, and nasal corneal quadrants which were covered by the pterygium before surgery as well as the corneal center (Figure 1) were evaluated using perpendicular contacts by applying the ascending method of limits, starting with a length of 60 mm and decreasing in steps of 5 mm. Two positive responses in three attempts at each filament length were regarded as the threshold to stimulation. The results are presented as millimeters of nylon filament length.

### 2.6. *In Vivo* Confocal Microscopy

All patients underwent IVCM analysis using the Rostock Cornea Module of the Heidelberg Retina Tomograph II (HRT II RCM) before surgery as well as 1 month and 3 months after surgery. IVCM was also performed in all healthy controls. Before the IVCM examination, one drop of topical anesthetic (benoxil 0.4%; Santen Pharmaceutical Co. Ltd., Japan) was administered. Each frame consisted of 384 × 384 pixels covering an area of 400 × 400 *μ*m with a transversal optical resolution of 0.96 pixel/*μ*m and an acquisition time of 0.024 seconds (Heidelberg Engineering GmbH, Heidelberg, Germany). Special attention was given to the subbasal nerve plexus layer to evaluate the nerve plexuses. Three representative images of the subbasal nerve plexus at the central cornea were selected in the analysis of each eye considering criteria such as the whole image in the same layer, best focus, good contrast, and most nerve fibers.

**Figure 2:**
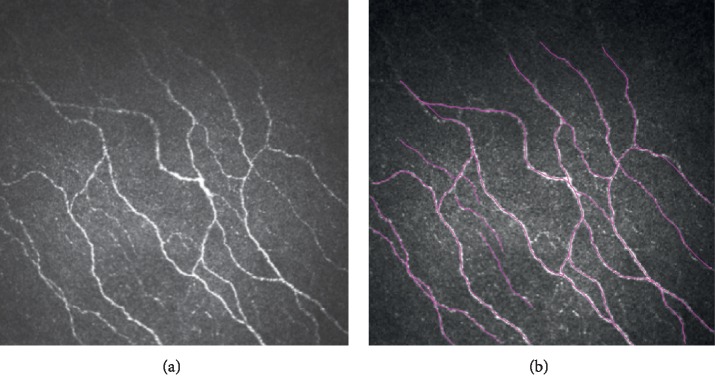
Analysis of *in vivo* confocal microscopy images (400 × 400 *μ*m) of corneal subbasal nerves using the semiautomatic tracing program of NeuronJ software.

The analysis of the subbasal nerve plexus was performed using the semiautomated tracing program NeuronJ (Biomedical Imaging Group, USA), as described previously [22]. The entire frame was analyzed, and the nerve density was obtained by measuring the total length of the nerve fibers per frame, as shown in Figure 2. Adjacent layers were compared to ensure that the main nerve trunks did not branch from other nerves, and the total number of the main nerve trunks was then counted manually in each image. Morphologically, nerve trucks appeared to be thick, straight, and highly reflective in HRT scans with few nerve branches while nerve branches were thin, tortuous, and less reflective. The total number of nerve branches was also calculated in each image. Nerve tortuosity was graded according to criteria described by Oliveira-Soto and Efron [23]. Two masked observers (Z.Z. and J.Z.) evaluated the corneal subbasal nerve plexus independently using the same criteria, and data were then statistically analyzed.

### 2.7. Statistical Analysis

All data of patients and controls passed the test of normality (Shapiro–Wilk test), indicating Gaussian distribution. In GraphPad Prism version 6.00 for Mac software, the unpaired *t*-test was used to compare results between controls and patients. A *P* value <0.05 using one-way repeated measures ANOVA (GraphPad Prism version 6.00 for Mac) was considered statistically significant in the comparisons of preop, 10 d postop, 1 m postop, and 3 m postop data. Pearson correlation (GraphPad software) was used to calculate whether the pterygium-affected corneal area correlated with the difference between the pre- and postoperative nerve densities. Data were expressed as mean ± standard error of the mean.

## 3. Results

A total of 22 eyes of 17 patients with primary pterygium (five men and 12 women) aged 47–69 years (median: 58 years) were included in the present study, along with 18 eyes from 18 healthy participants (eight men and 10 women) aged 44–73 years (median: 62 years). The percentage of corneal area covered by the pterygium ranged from 4.07% to 19.88% with a median of 10.24%.

### 3.1. Results of Schirmer's Test and TBUT Test

**Figure 3:**
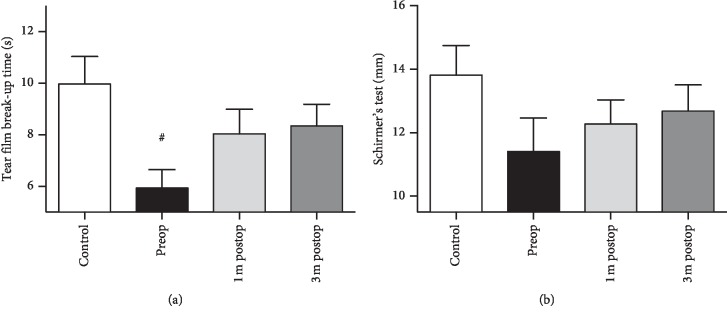
Results of tear film break-up time (TBUT) test (a) and Schirmer's test (b) in healthy controls and pterygium patients. ^#^*P* < 0.05 compared with normal controls.

Corroborating the idea [14] that abnormal growth and proliferation of cells onto the peripheral cornea can disturb the tear film, contributing to the dry eye, pterygium patients demonstrated lower TBUT than controls (5.93 ± 0.72 seconds vs. 9.95 ± 1.07 seconds; *P* < 0.01). Affected eyes showed a tendency toward increased TBUTafter surgery (preop, 1 m postop, and 3 m postop: 5.93 ± 0.72 seconds, 8.03 ± 0.95 seconds, and 8.34 ± 0.84 seconds, respectively; *F* = 2.87, *P*=0.07; Figure 3(b)).

No difference was observed between the results of Schirmer's test. Control eyes had a value of 13.81 ± 0.93 mm, while patients' eyes had a value of 11.41 ± 1.06 mm before surgery. The postop values were 12.27 ± 0.76 mm and 12.68 ± 0.83 mm, respectively, in the patient group 1 month and 3 months after surgery (*F* = 0.82, *P* > 0.05; Figure 3(a)).

### 3.2. Improved Sensitivity of Nasal Corneal Quadrant after Surgery

The average corneal sensitivity values in the quadrants of the control eyes were as follows: nasal, 58.06 ± 1.67 mm; superior, 54.17 ± 1.53 mm; temporal, 60 ± 0 mm; inferior, 58.33 ± 1.14 mm; and central cornea, 59.72 ± 0.28 mm. All affected eyes in patients showed deteriorated corneal sensitivity in the nasal quadrant when compared with controls (42.61 ± 2.21 mm vs. 58.06 ± 1.67 mm, respectively; *P* < 0.01).

**Figure 4:**
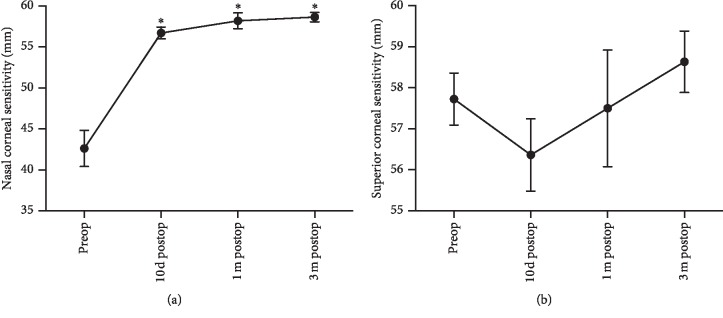
Restored sensitivity was observed on the nasal corneal quadrant after surgery (a), and superior corneal sensitivity dropped insignificantly 10 d postop (b). ^*∗*^*P* < 0.01 compared with preop value.

In addition, patients showed a significant increase in sensitivity in the nasal corneal quadrant at 10 d postop, 1 m postop, and 3 m postop when compared with preop data (preop, 10 d postop, 1 m postop, and 3 m postop: 42.61 ± 2.21 mm, 56.70 ± 0.72 mm, 58.18 ± 0.96 mm, and 58.64 ± 0.59 mm, respectively; *F* = 37.3, *P* < 0.01; Figure 4(a)). Superior corneal sensitivity dropped after surgery although not by a statistically significant amount (preop, 10 d postop, 1 m postop, and 3 m postop values: 57.73 ± 0.64 mm, 56.36 ± 0.88 mm, 57.50 ± 1.43 mm, and 58.64 ± 0.75 mm, respectively; *F* = 0.95, *P* > 0.05; Figure 4(b)).

The inferior and temporal quadrants of the cornea did not differ significantly from the corneal center in either the pre- or postop corneal sensitivity data (Table 1).

### 3.3. Deteriorated Corneal Nerve Fibers in Patients with Pterygium and Central Corneal Nerve Fiber Regeneration after Pterygium Excision

The whorl-like form of the corneal nerve plexus is different in each individual, and the subbasal nerve density depends on the location within the cornea [31], so we conducted IVCM in the central cornea to minimize bias.

**Figure 5:**
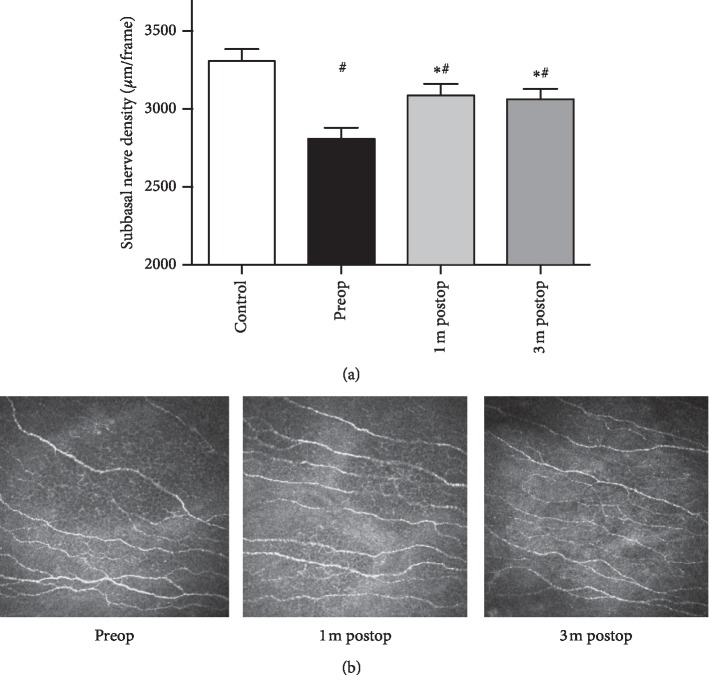
Pterygium patients presented a lower subbasal nerve density than healthy controls, whereas a greater central subbasal nerve density was observed 1 month and 3 months after surgery (a). Representative *in vivo* confocal microscopy frames from one patient (b).

Patients with pterygium demonstrated less corneal subbasal nerve density than controls (2810 ± 70.68 *μ*m/frame vs. 3307 ± 76.77 *μ*m/frame, respectively; *P* < 0.01; Figure 5(a)). Patients with pterygium also had fewer nerve trunks (2.322 ± 0.1422 vs. 2.981 ± 0.1058; *P* < 0.01) and fewer nerve branches (7.936 ± 0.2876 vs. 9.440 ± 0.5790; *P* < 0.05) (Table 2). Nerve tortuosity showed no significant difference between pterygium-affected eyes and controls (3.023 ± 0.1271 vs. 2.995 ± 0.1645; *P* > 0.05).

Compared with preoperative central corneal subbasal nerve density, the values at 1 m postop and 3 m postop were significantly higher (2810 ± 70.68 *μ*m/frame, 3087 ± 73.80 *μ*m/frame, and 3061 ± 68.14 *μ*m/frame, respectively; *F* = 9.62, *P* < 0.01; Figure 5(a)).  Figure 5(b) shows representative IVCM frames of the central cornea from one patient. The difference in central corneal subbasal nerve densities between 1 m postop and 3 m postop was not significant, suggesting that the central corneal subbasal nerve plexus had recovered by 1 month after surgery. Similarly, nerve trunks, nerve branches, and nerve tortuosity showed no significant change after surgery in patients (Table 2).

Pterygium area showed no correlation with central nerve growth or increased sensitivity in the nasal corneal quadrant.

**Figure 6:**
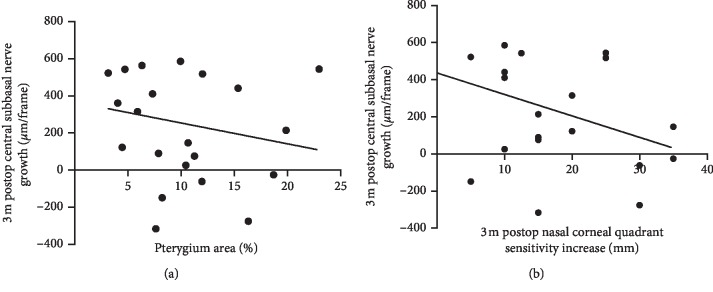
No signficant correlation was found between pterygium area and 3 m postop central subbasal nerve growth (a) or between pterygium area and 3 m postop increase in nasal corneal quadrant sensitivity (b).

Based on the confocal microscopy data, the pterygium area showed no linear correlation with 3 m postop central subbasal nerve growth (*R* = −0.26; *P*=0.24; Figure 6(a)). Furthermore, no correlations were found between sensitivity increase and central nerve density growth (*R* = −0.40; *P*=0.07; Figure 6(b)).

## 4. Discussion

Our study found deteriorated corneal subbasal nerve fibers and unstable tear film in patients with pterygium when compared with healthy eyes. In contrast, we observed increased central subbasal nerve density along with better corneal sensitivity 1 month and 3 months after pterygium excision. This suggests that the functional sensitivity changes are related to anatomical corneal nerve changes in those patients.

The cornea is a highly innervated structure. In addition to their important sensory function, corneal nerves provide protective and trophic functions, and they regulate corneal epithelial integrity, proliferation, and wound healing. Loss of corneal nerves can lead to neurotrophic keratitis and corneal anesthesia [19]. Thus, corneal sensitivity is a necessary component of corneal maintenance. Partial or complete corneal anesthesia may indicate neural damage before clinically detectable lesions are observed [17]. Previous studies have shown that pterygium can also lead to partial anesthesia of affected corneas [16]. Therefore, we evaluated both corneal sensitivity and subbasal nerve structures before and after pterygium excision.

We found that the sensitivity of the nasal corneal quadrant in patients with pterygium dropped to about 42.61 ± 2.21 mm, while the rest of the corneas remained normal or only slightly altered, corroborating previous studies [15, 16, 32]. In two previous studies, decreased corneal sensitivity was related to altered structure in Bowman's membrane, where no nerve plexus could be detected using IVCM [24, 25]. The decrease in corneal sensitivity in patients with pterygium may also involve neural damage caused by ultraviolet light [2] and/or chronic inflammation [24, 25] in the cornea and conjunctiva.

IVCM is a noninvasive imaging technique that allows detailed quantification of the corneal subbasal nerve plexus. Recent IVCM studies in contact lens wearers [33], patients who had undergone LASIK [21, 34], and those with diabetes[35] have documented loss of the subbasal nerve plexus and the regenerative capacity of corneal nerves. To minimize bias caused by eye movements during confocal microscopy and to consider the effects of surgery on the entire ocular surface, we evaluated the subbasal nerve density in the central cornea. Pterygium patients demonstrated fewer corneal subbasal nerve fibers, fewer nerve trunks, and fewer nerve branches than controls perhaps because pterygium invades Bowman's membrane at the corneal limbus, deteriorating the corneal subbasal nerves [23, 24]. The central corneal nerve fibers had already regenerated 1 month after pterygium excision and become stable 3 months after surgery. Procedures such as cataract surgery [36] and corneal refractive surgery [37] lead to degraded nerve fibers. In contrast, after pterygium excision, the central subbasal corneal nerve fibers may rapidly regenerate because the surgical incisions are mainly made in the conjunctival and epithelial layers rather than the scleral layer or corneal stroma. This leaves the main corneal nerve trunks intact as the long ciliary nerves from the nasociliary branch of V1 pass through the sclera and transmit sensory fibers to the cornea.

The relationship between corneal nerve function and structure remains complex and varies across different ocular surface diseases [17, 21, 22]. In patients with dry eye, higher tortuosity and more beadings are considered signs of high metabolic activity in response to epithelial alterations [38]. This study did not demonstrate any difference in corneal tortuosity between healthy eyes and patients with pterygium. Nevertheless, we did find restored corneal sensitivity 10 days after surgery, indicating that sensitivity could be recovered fast. Further study of subbasal corneal nerve morphological changes is needed.

Pterygium may lead to decreased tear film stability due to the epithelial irregularities and chronic inflammation. We found improved TBUT results after pterygium excision and a tendency of improvement in tear secretion. These findings along with the improved corneal sensitivity and reinnervation after pterygium excision may suggest that the increased nerve fibers might play a better role in corneal nutrition and corneal reflex, contributing to the homeostasis of the ocular surface.

In summary, the present findings helped us understand corneal nerve alternations during pterygium. We found corneal reinnervation along with improved sensitivity in the nasal corneal quadrant after pterygium excision in patients with primary pterygium. The restored corneal sensitivity and ocular defense mechanisms protect the ocular surface and vision in patients with previously hypoesthetic corneas. Biomolecular study of sensory neuropeptides (substance P, calcitonin gene-related peptide, and nerve growth factor) and inflammatory cytokines should be carried out to explore the pathophysiological molecular mechanisms involved in this phenomenon.

## Figures and Tables

**Table 1:** Corneal sensitivity in the nasal, superior, temporal, inferior, and central quadrants in both controls and pterygium patients.

Cochet–Bonnet esthesiometer results	Control (mm)	Preop (mm)	10 d postop (mm)	1 m postop (mm)	3 m postop (mm)	*F*	*P* value
Nasal	58.06 ± 1.67	42.61 ± 2.21	56.70 ± 0.72	58.18 ± 0.96	58.64 ± 0.59	37.03	<0.01
Superior	54.17 ± 1.53	57.73 ± 0.64	56.36 ± 0.88	57.50 ± 1.43	58.64 ± 0.75	0.95	0.40
Temporal	60 ± 0	59.55 ± 0.31	60 ± 0	59.55 ± 0.45	60 ± 0	1.15	0.32
Inferior	58.33 ± 1.14	59.77 ± 0.23	58.86 ± 0.46	59.55 ± 0.31	59.32 ± 0.37	1.14	0.34
Central	59.72 ± 0.28	60 ± 0	60 ± 0	60 ± 0	60 ± 0	—	—

**Table 2:** Corneal subbasal nerve parameters in patients and controls.

Parameters	Control	Preop	1 m postop	3 m postop	*F*	*P* value
Nerve trunks (*n*/frame)	2.981 ± 0.1058	2.322 ± 0.1422^##^	2.496 ± 0.1553^#^	2.337 ± 0.1954^##^	1.05	0.35
Nerve branches (*n*/frame)	9.440 ± 0.5790	7.936 ± 0.2876^#^	7.905 ± 0.3373^#^	7.867 ± 0.3539^#^	0.02	0.98
Nerve tortuosity	2.995 ± 0.1645	3.023 ± 0.1271	3.017 ± 0.09255	3.089 ± 0.1250	0.21	0.80

^#^ *P* < 0.05 compared with controls, ^##^*P* < 0.01 compared with controls.

## Data Availability

Data are available under considerable request.
